# Ethyl Acetate Fraction from Persimmon (*Diospyros kaki*) Ameliorates Cerebral Neuronal Loss and Cognitive Deficit via the JNK/Akt Pathway in TMT-Induced Mice

**DOI:** 10.3390/ijms19051499

**Published:** 2018-05-17

**Authors:** Jong Min Kim, Seon Kyeong Park, Jin Yong Kang, Su Bin Park, Seul Ki Yoo, Hye Ju Han, Chul-Woo Kim, Uk Lee, Sea-Hyun Kim, Ho Jin Heo

**Affiliations:** 1 Division of Applied Life Science (BK21 plus), Institute of Agriculture and Life Science, Gyeongsang National University, Jinju 52828, Korea; myrock201@naver.com (J.M.K.); tjsrud2015@naver.com (S.K.P.); kangjy2132@naver.com (J.Y.K.); tbsk5670@naver.com (S.B.P); ysyk9412@naver.com (S.K.Y.); gksgpwn2527@naver.com (H.J.H.); 2 Division of Special Forest Products, National Institute of Forest Science, Suwon 16631, Korea; futuretree@korea.kr (C.-W.K.); rich26@korea.kr (U.L.); goldtree@korea.kr (S.-H.K.)

**Keywords:** *Diospyros kaki*, neuroprotective effect, trimethyltin, cognitive function, JNK/Akt pathway

## Abstract

This study was conducted to assess the antioxidant capacity and protective effect of the ethyl acetate fraction from persimmon (*Diospyros kaki*) (EFDK) on H_2_O_2_-induced hippocampal HT22 cells and trimethyltin chloride (TMT)-induced Institute of Cancer Research (ICR) mice. EFDK had high antioxidant activities and neuroprotective effects in HT22 cells. EFDK ameliorated behavioral and memory deficits in Y-maze, passive avoidance and Morris water maze tests. Also, EFDK restored the antioxidant system by regulating malondialdehyde (MDA), superoxide dismutase (SOD) and reduced gluthathione (GSH), and the cholinergic system by controlling the acetylcholine (ACh) level and acetylcholinesterase (AChE) activity and expression. EFDK enhanced mitochondrial function by regulating reactive oxygen species (ROS) production, mitochondrial membrane potential (MMP), and adenosine triphosphate (ATP). Ultimately, EFDK regulated the c-Jun N-terminal kinase (JNK)/protein kinase B (Akt) pathway and apoptotic pathway by suppressing the expression of tumor necrosis factor-alpha (TNF-α), phosphorylated insulin receptor substrate 1 (IRS-1pSer), phosphorylated JNK (p-JNK), phosphorylated tau (p-tau), phosphorylated nuclear factor kappa-light-chain-enhancer of activated B cells (p-NF-κB), Bcl-2-associated X protein (BAX) and cytosolic cytochrome c, and increasing the expression of phosphorylated Akt (p-Akt) and mitochondrial cytochrome c. This study suggested that EFDK had antioxidant activity and a neuroprotective effect, and ameliorated cognitive abnormalities in TMT-induced mice by regulating the JNK/Akt and apoptotic pathway.

## 1. Introduction

Alzheimer’s disease (AD), one of the neurodegenerative diseases, is associated with the death of brain neuronal cells that affect learning and cognitive function [1]. Oxidative stress, one of the causes of AD, is caused by the dysfunction of the system that eliminates the toxicity of products such as reactive oxygen species (ROS) [2]. This stress leads to damage to cerebral neurons, especially those with high oxygen consumption and low levels of antioxidants [3]. Also, neurons contain a large amount of unsaturated fatty acids that can react with ROS, leading to continuous lipid peroxidation and cell damage [4]. This oxidative stress promotes the production of amyloid plaque and increases the phosphorylation of tau protein as an ingredient of neurofibrillary tangles (NFTs) in cerebral nervous tissue. This accumulation of amyloids and NFTs induces the death of neuronal cells [5]. It also causes damage to nerve cells through sustained inflammatory responses. Such cell damage occurs at sites such as the hippocampus, amygdala, frontal cortex, and thalamus of brain tissue, and shows a decrease in volume and weight of brain tissue. This cell death ultimately leads to cognitive dysfunction and expression of AD [2]. Interest in various antioxidants to reduce these stresses has increased. These antioxidants are known to regulate the redox reaction in neurons by eliminating the free radicals of ROS and reducing oxidative stress [6].

Persimmon (*Diospyros kaki*) is widely distributed in East Asia, such as Korea, Japan, and China. Persimmon contains a variety of phytochemicals, known as proanthocyanidins, flavonoid oligomers, tannins, phenolic acids and carotenoids [7]. In particular, the pulp contains various polyphenol compounds such as catechin, caffeic acid, chlorogenic acid, ferulic acid, gallic acid, rutin, and vanillic acid [8]. The physiological activities of persimmon are related to previously mentioned bioactive compounds that have a protective effect against metabolic diseases, cancer, atherosclerosis and hypertension [9]. Many studies have reported that persimmon, which contains various phenolic compounds, has anti-inflammatory, anticarcinogenic, and antimutagenic effects based on its high antioxidant effect [10]. It also contains a large amount of tannin. This tannin, which acts as a fibrin, combines with bile acids to improve cholesterol [11]. Although persimmon contains a variety of physiologically active substances, research on the protective effect on cognitive function is lacking. Thus, this study aimed at investigating the ameliorating effect of ethyl acetate fraction from persimmon (EFDK) in trimethyltin chloride (TMT)-induced Institute of Cancer Research (ICR) mice by conducting an in vivo test, biochemical assay and signaling analysis related to oxidative stress and AD pathology.

TMT is an organotin compound with potent neurotoxicity and is known to cause significant damage to the hippocampus in the brains of rodents [12]. After treatment with TMT, mice show damage to pyramidal neurons in the hippocampal CA3 region of the brain [13]. TMT-induced hippocampal damage results in behavioral and learning disabilities such as hyperactivity. Although the molecular mechanism of TMT is not fully understood, it is generally accepted that TMT induces oxidative stress and damages cranial nerves [14]. Thus, it is a useful model of neurodegeneration and death because it exhibits neurodegeneration, epilepsy and neurobehavioral disorders [15].

## 2. Results

### 2.1. Antioxidant Capacity

To assess the antioxidant activity of EFDK, total phenolic contents (TPC), total flavonoid contents (TFC), ABTS and DPPH radical scavenging activities and malondialdehyde (MDA) inhibitory effect were ascertained (Table 1). TPC and TFC were assigned to compare to the gallic acid and Rutin standard curve. EFDK had considerable contents of phenolic and flavonoid compound (81.75 mg of GAE/g and 187.52 mg of RE/g). It also had high ABTS scavenging activity (IC_50_ value: 25.03 μg/mL when compared to the IC_50_ value of vitamin C (4.15 μg/mL) as positive control) and DPPH (IC_50_ value: 35.47 μg/mL when compared to the IC_50_ value of vitamin C (2.56 μg/mL) as positive control), and an inhibitory effect on MDA (IC_50_ value: 389.21 μg/mL when compared to the IC_50_ value of catechin (22.30 μg/mL) as positive control).

### 2.2. Protective Effect on H_2_O_2_-Induced Neurotoxicity in Hippocampal HT22 Cells

**Figure 1:**
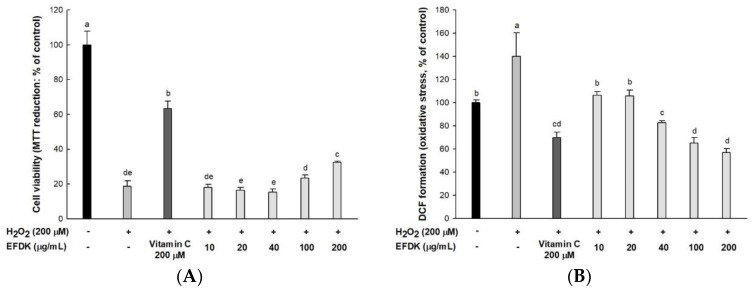
Neuroprotective effect of ethyl acetate fraction from persimmon (EFDK) in HT22 cells. (**A**) Cell viability; (**B**) cellular oxidative stress. Results shown are mean ± SD (*n* = 3). Data were statistically represented at *p* < 0.05, and different small alphabets mean statistical significance.

To evaluate the neuroprotective effect of EFDK, cell viability and intracellular ROS were measured using MTT and DCF-DA analysis in hippocampal HT22 cells (Figure 1). The cell viability of the H_2_O_2_-treated group (18.66%) was reduced compared to the control group (100%). However, that of EFDK-treated groups showed increased cell viability (200 μg/mL, 32.40%; 100 μg/mL, 23.53%, respectively) compared to the H_2_O_2_-treated group. By contrast, intracellular ROS in the H_2_O_2_-treated group (140.04%) increased compared to the control group (100%). However, the ROS production of the EFDK-treated groups (200 μg/mL, 56.85%; 100 μg/mL, 65.26%, respectively) was reduced compared to that of the H_2_O_2_-treated group.

### 2.3. Y-Maze Test and Passive Avoidance Test

**Figure 2:**
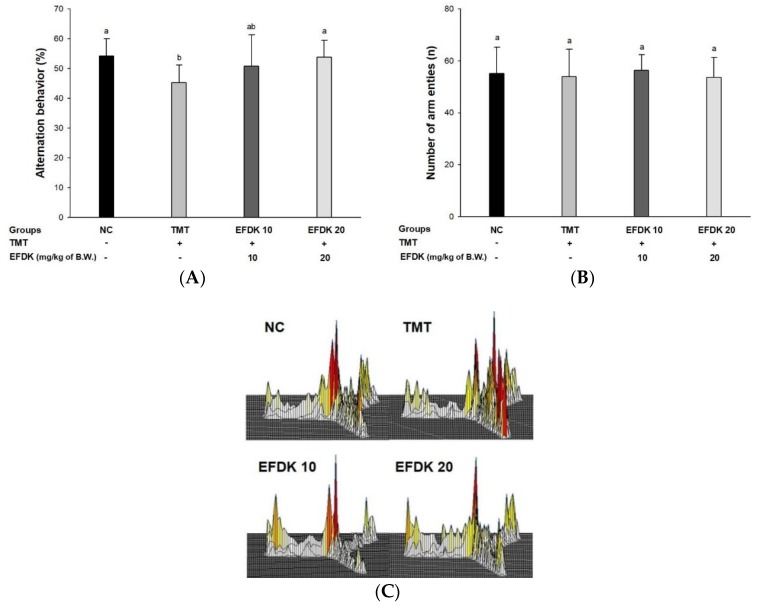
Protective effect of ethyl acetate fraction from persimmon (EFDK) on Y-maze test in TMT-induced mice. (**A**) Spontaneous alternation behavior; (**B**) number of arm entries; (**C**) 3D moving routes. Results shown are mean ± SD (*n* = 8). Data were statistically represented at *p* < 0.05, and different small alphabets mean statistical significance.

To evaluate the alternation behavioral function of EFDK against TMT-induced oxidative stress, a Y-maze test was conducted (Figure 2). The spontaneous alternation behavior of the TMT group (45.27%) decreased significantly compared to the NC group (54.16%) (Figure 2A). On the other hand, the EFDK groups (50.76% and 53.80%, respectively) indicated improved spontaneous alternation behavior compared to the TMT group. It was confirmed that there was no problem in exercise capacity by observing that there was no difference in the number of movements (Figure 2B). The TMT group showed irregular movement compared to the NC group. On the other hands, that of the EFDK groups was similar to the NC group (Figure 2C).

### 2.4. Passive Avoidance Test

**Figure 3:**
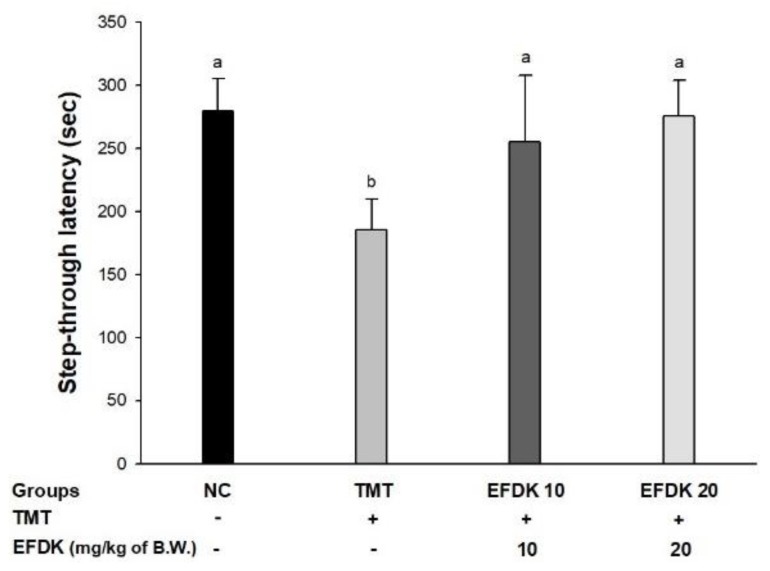
Protective effect of effect of ethyl acetate fraction from persimmon (EFDK) on passive avoidance test in TMT-induced mice. Results shown are mean ± SD (*n* = 8). Data were statistically represented at *p* < 0.05, and different small alphabets mean statistical significance.

To assess short-term memory, a passive avoidance test was conducted (Figure 3). The latency time of the TMT group (185.50 s) decreased compared to the NC group (279.57 s). Whereas, the EFDK groups (255.33 s and 275.67 s, respectively) showed ameliorated short-term memory ability compared to the TMT group. 

### 2.5. Morris Water Maze Test

**Figure 4:**
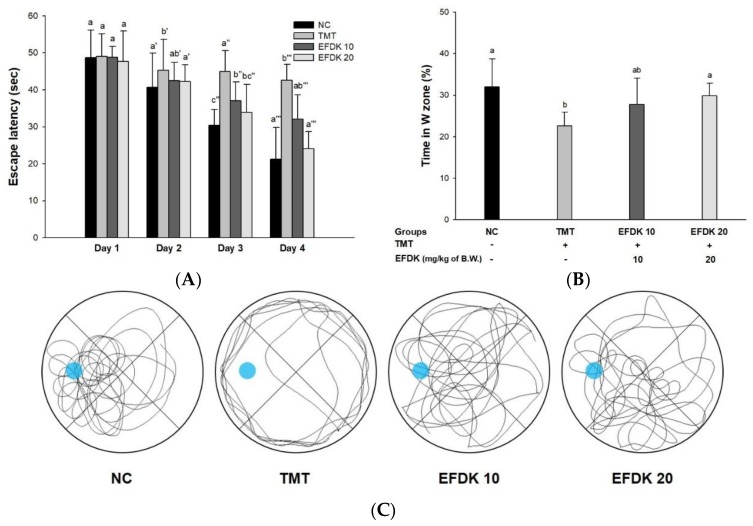
Protective effect of ethyl acetate fraction from persimmon (EFDK) in the Morris water maze test in TMT-induced mice. (**A**) Escape latency in the training trial; (**B**) retention time on W zone in the probe trial; (**C**) path tracing of each groups in the probe trial. Results shown are mean ± SD (*n* = 8). Data were statistically represented at *p* < 0.05, and different small alphabets mean statistical significance.

To estimate spatial learning acquisition and long-term memory, a Morris water maze test was conducted (Figure 4). In the hidden trial (Figure 4A), the latency time to escape to the platform was gradually reduced in all of the groups. In the last session, the latency time of the TMT group (42.62 s) was diminished more than it was for the NC group (21.22 s), while the EFDK groups (32.11 s and 24.04 s, respectively) indicated reduced time to escape. In the probe test (Figure 4B), the latency time of the TMT group in the W zone where the platform was (22.62%) was reduced compared to the NC group (32.04%). However, the EFDK-treated groups (27.78% and 29.82%, respectively) showed increasing time in the W zone. Comparing tracked movements in the W zone, the movements of the TMT group were not concentrated in the target zone like those of the NC group (Figure 4C). The EFDK groups showed more ameliorated movement than the TMT group.

### 2.6. Ferric-Reducing Ability of Plasma (FRAP) and Antioxidant System in Brain Tissue

**Figure 5:**
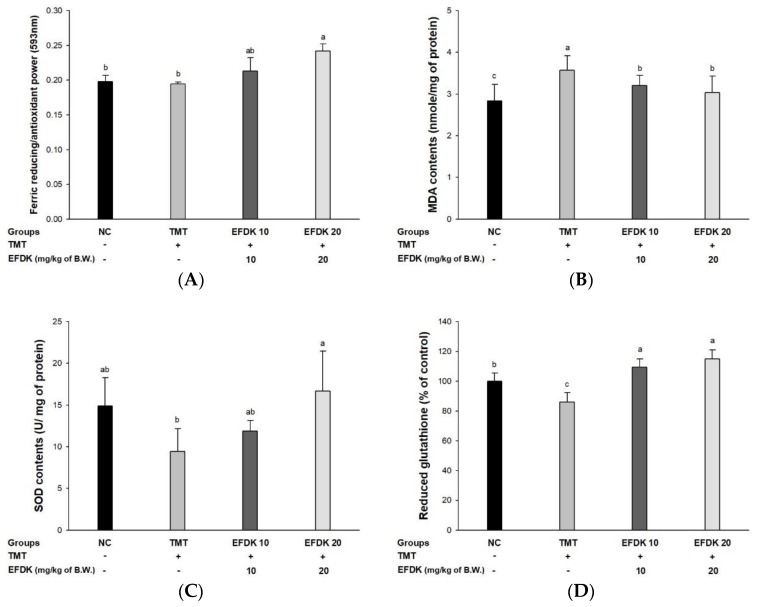
Protective effect of ethyl acetate fraction from persimmon (EFDK) on TMT-induced biochemical changes related with antioxidant system. (**A**) Plasma ferric-reducing ability of plasma (FRAP); (**B**) MDA levels; (**C**) superoxide dismutase (SOD) contents; (**D**) reduced gluthathione (GSH) levels in mice brain tissues. Results shown are mean ± SD (*n* = 8). Data were statistically represented at *p* < 0.05, and different small alphabets mean statistical significance.

A ferric-reducing ability of plasma (FRAP) assay was performed to measure the antioxidant activity of the plasma of EFDK-treated mice (Figure 5A). There was no difference in plasma FRAP between the NC group (0.19) and the TMT group (0.19). However, the EFDK groups increased plasma FRAP to 0.21 and 0.24, respectively. 

To examine the antioxidant system with the consumption of EFDK, MDA level, superoxide dismutase (SOD) content and reduced glutathione (GSH) activity were measured (Figure 5B–D). The MDA level of TMT (3.57 nmole/mg of protein) increased compared to the NC group (2.83 nmole/mg of protein) (Figure 5B). However, that of the EFDK groups (3.20 nmole/mg of protein and 3.04 nmole/mg of protein) were reduced compared to the TMT group. The SOD contents (Figure 5C) and reduced GSH level (Figure 5D) of the TMT group (9.41 U/mg of protein and 86.04%) decreased compared to the NC group (14.87 U/mg of protein and 100%). However, the EFDK groups showed increased SOD contents (11.89 U/mg of protein and 16.66 U/mg of protein) and reduced GSH activity (109.46% and 115.02%) compared to the TMT group.

### 2.7. Cholinergic System in Brain Tissue

**Figure 6:**
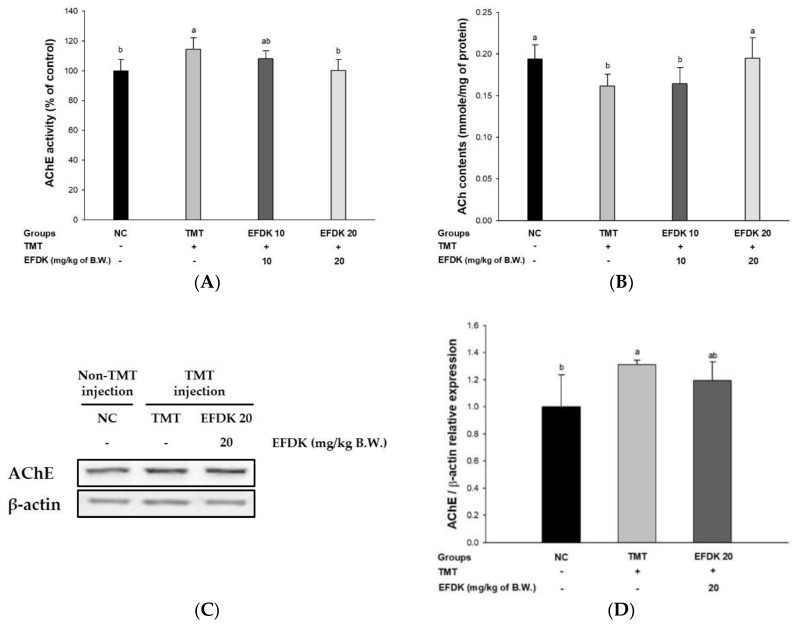
Protective effect of ethyl acetate fraction from persimmon (EFDK) on TMT-induced cholinergic dysfunction. (**A**) AChE activity; (**B**) ACh level; (**C**) representative western blots for total protein and expression of AChE; (**D**) protein expression levels of AChE normalized to β-actin. Results shown are mean ± SD ((**A**,**B**), *n* = 8; (**C**,**D**), *n* = 3). Data were statistically represented at *p* < 0.05, and different small alphabets mean statistical significance.

In order to estimate the protective effect of the cholinergic system, acetylcholinesterase (AChE) activity and acetylcholine (ACh) content were examined (Figure 6). The TMT group showed more activated AChE (114.34%) (Figure 6A) and reduced ACh level (0.16 mmole/mg of protein) (Figure 6B) than the NC group (100% and 0.19 mmole/mg of protein, respectively). On the other hand, the EFDK groups presented inhibited AChE activities (108.12% and 100.10%, respectively) and increased ACh contents (0.16 mmole/mg of protein and 0.19 mmole/mg of protein, respectively) (Figure 6D). Also, the expression of AChE of TMT (31.09%) was up-regulated more than it was for the NC group. However, the EFDK groups presented a down-regulated expression of AChE (8.96%) in comparison with the TMT group.

### 2.8. Cerebral Mitochondrial Function

**Figure 7:**
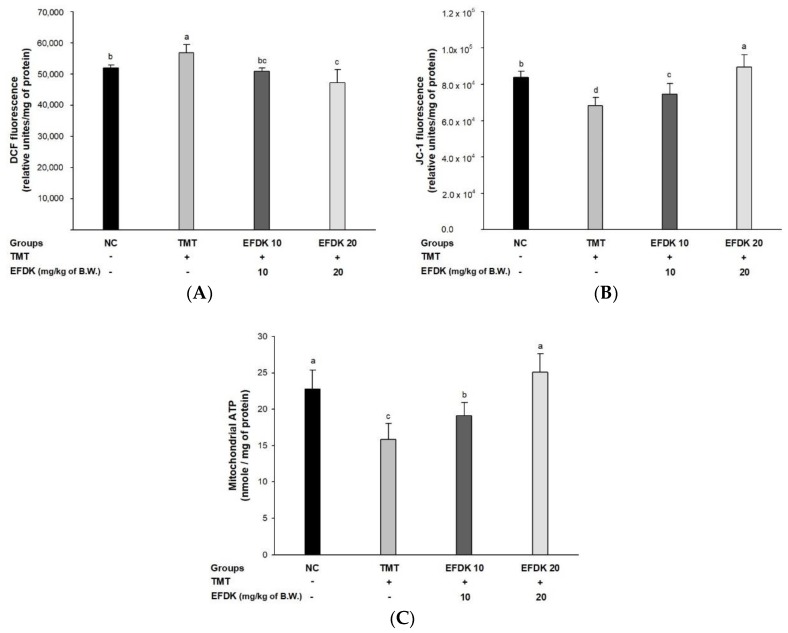
Protective effect of ethyl acetate fraction from persimmon (EFDK) on TMT-induced mitochondrial dysfunction. (**A**) ROS levels; (**B**) MMP levels; (**C**) ATP contents of the mitochondria in mice brain tissues. Results shown are mean ± SD (*n* = 5). Data were statistically represented at *p* < 0.05, and different small alphabets mean statistical significance.

To assess the ameliorating effect of EFDK on TMT-induced abnormal mitochondrial function, ROS levels, mitochondrial membrane potential (MMP) and ATP contents were measured (Figure 7). The ROS contents of the TMT group (56,889.48 relative units/mg of protein) were increased in comparison with the NC group (52,020.84 relative units/mg of protein) (Figure 7A), while that of the EFDK group (50,877.63 relative units/mg of protein and 47,248.46 relative units/mg of protein, respectively) was reduced compared to the TMT group. Also, the MMP and ATP contents (68,371.04 relative units/mg of protein and 15.80 nmole/mg of protein, respectively) of the TMT group were diminished more than for the NC group (83,727.63 relative units/mg of protein and 22.76 nmole/mg of protein) (Figure 7B,C). However, the EFDK groups presented ameliorated MMP (74,637.97 relative units/mg of protein and 89,486.40 relative units/mg of protein) and ATP contents (19.12 nmole/mg of protein and 25.18 nmole/mg of protein, respectively).

### 2.9. Neuronal JNK/Akt Pathway

**Figure 8:**
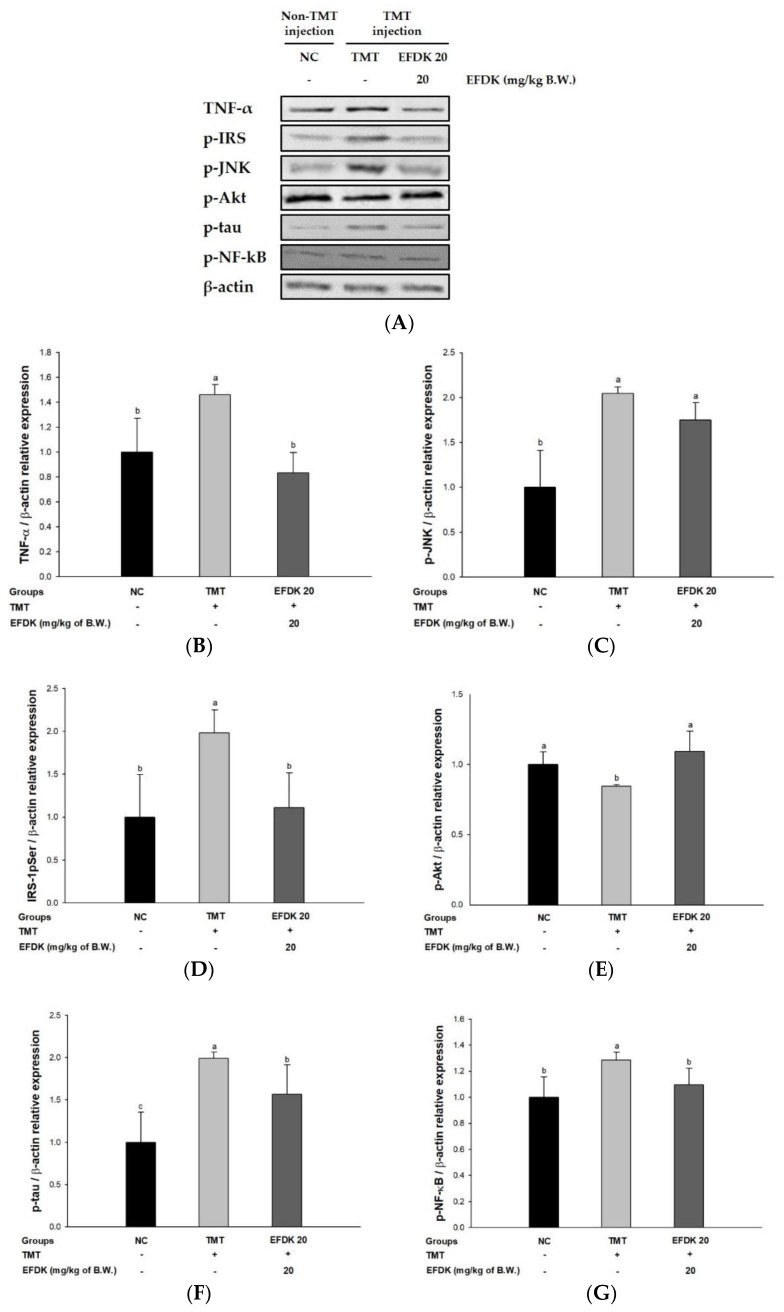
Protective effect of ethyl acetate fraction from persimmon (EFDK) on JNK/Akt pathway in mice brain tissues. (**A**) Representative western blots for total protein and expression of TNF-α, p-JNK, IRS-1pSer, p-Akt, p-tau, p-NF-κB and β-actin; Protein expression levels of TNF-α (**B**); p-JNK (**C**); IRS-1pSer (**D**); p-Akt (**E**); p-tau (**F**) and p-NF-κB (**G**) normalized to β-actin. Results shown are mean ± SD (*n* = 3). Data were statistically represented at *p* < 0.05, and different small alphabets mean statistical significance.

To investigate the regulating effect of the c-Jun N-terminal kinase (JNK)/protein kinase B (Akt) signaling pathway, western blotting was carried out (Figure 8). The expression of tumor necrosis factor-alpha (TNF-α), phosphorylated JNK (p-JNK), phosphorylated insulin receptor substrate-1 (IRS-1pSer), phosphorylated tau (p-tau) and phosphorylated nuclear factor kappa-light-chain-enhancer of activated B cells (p-NF-κB) of the TMT group (46.14%, 104.36%, 98.14%, 99.30% and 28.68%, respectively) increased more than for the NC group. However, the EFDK groups showed down-regulated levels of TNF-α, p-JNK, IRS-1pSer, p-tau and p-NF-κB (42.87%, 14.36%, 44.02%, 21.31% and 14.81%, respectively) compared to the TMT group. In addition, the expression of p-Akt of the TMT group (15.61%) decreased more than for the NC group. But the p-Akt expression of the EFDK group (29.52%) was up-regulated more than for the TMT group.

### 2.10. Neuronal Apoptotic Pathway

**Figure 9:**
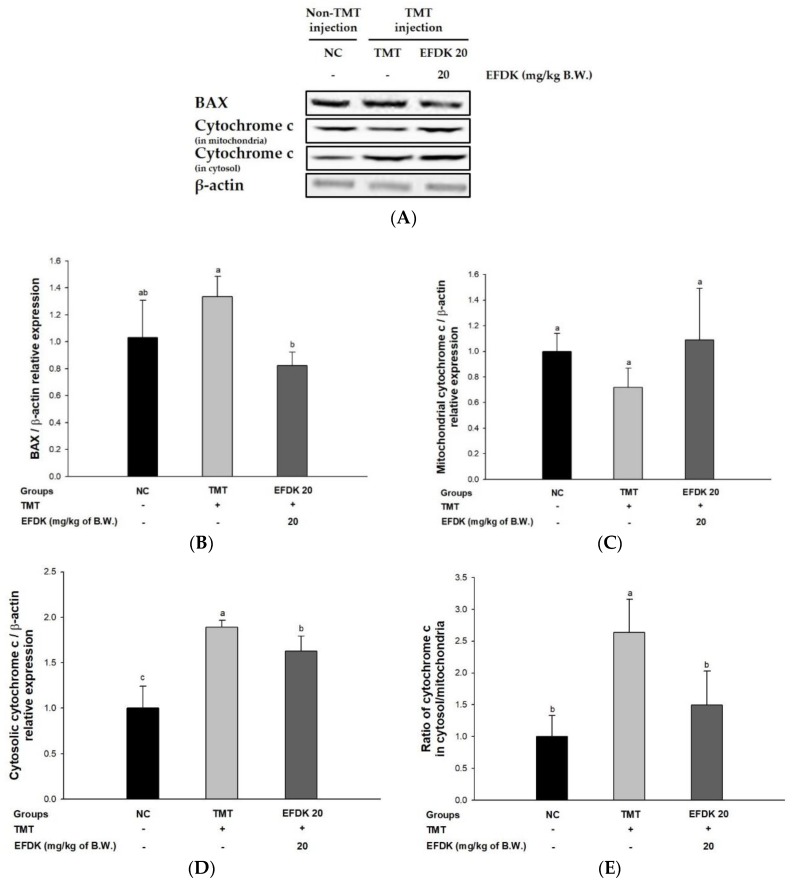
Protective effect of ethyl acetate fraction from persimmon (EFDK) on apoptotic pathway in mice brain tissues. (**A**) Representative Western blots for total protein and expression of BAX, mitochondrial cytochrome c, cytosolic cytochrome c and β-actin; Protein expression levels of BAX (**B**); mitochondrial cytochrome c (**C**); cytosolic cytochrome c (**D**) and ratio of cytochrome c in cytosol/mitochondria (**E**) normalized to β-actin. Results shown are mean ± SD (*n* = 3). Data were statistically represented at *p* < 0.05, and different small alphabets mean statistical significance.

In order to investigate the regulating effect of the apoptotic signaling pathway, Western blotting was carried out (Figure 9). The expression of Bcl-2-associated X protein (BAX) and cytosolic cytochrome c of the TMT group (29.31% and 88.85%, respectively) was increased compared to the NC group. However, those levels of the EFDK groups showed reduced expression of BAX and cytosolic cytochrome c (38.32% and 13.81%, respectively) compared to the TMT group. There was no significant difference in mitochondrial cytochrome c between all groups. However, the ratio of cytochrome c in cytosol/mitochondria in the TMT group increased (163.72%) compared to the NC group. The EFDK group showed a decreased ratio of cytochrome c in cytosol/mitochondria (43.28%) compared to the TMT group.

## 3. Discussion

AD is reported to be caused by neuronal diseases and degeneration due to oxidative stress [3]. Oxidative stress in AD patients’ brains is characterized by increased oxidation of proteins and DNA, lipid peroxidation, and decreased polyunsaturated fatty acids [2]. This stress also leads to a decrease in cerebral energy metabolism, and causes the formation of oxidation compounds such as aged glycation products, MDA, H_2_O_2_, and neurofibrillary tangles [3]. These results promote a decrease in antioxidants such as SOD and GSH in the brain, and induce amyloid-beta (Aβ) aggregation. This Aβ aggregation leads to neuronal death and ultimately to cognitive dysfunction and AD. TMT causes neural degeneration in brain tissue, particularly in the hippocampus region, and induces behavioral dysfunction, such as hyperactivity, cognitive impairment and memory loss [14]. These changes occur in the systemic damage of neurotransmitters such as GABA, glutamate, and acetylcholine by oxidative stress [16]. TMT inhibits GABA uptake by synaptosome in neurons. The inhibition of early GABA action leads to the gradual attenuation of neuronal activity. When the absorption of GABA is continuously suppressed, the extracellular concentration of GABA increases. Eventually, this leads to the depletion of GABA in synaptic vesicles, resulting in neurotransmitter abnormalities [12]. In a previous study, we analyzed the physiologically bio-active substances of ethanol extract of persimmon using high performance liquid chromatography (HPLC, U3000, Dionex, Sunnyvale, CA, USA) and ultra-performance liquid chromatography imaging mass spectrometry quadrupole time-of-flight mass spectrometry tandem mass spectrometry (UPLC-IMS-QTOF/MS^2^, Vion, Waters Corp., Milford, MA, USA), and these materials were identified as ascorbic acid (Table S1), glycerophosphocholine, adenosine, adenosine derives, gallic acid, pantothenic acid, lysophosphatidylcholine (LPC, C16:1), lysophosphatidylcholine (LPC, C18:1) and linolenic acid (Figures S1–S3). Therefore, in this study, we investigated the neuroprotective potential of EFDK containing the physiological compounds against neuronal cell death and cognitive dysfunction using H_2_O_2_ and TMT, which induce oxidative stress.

Oxidative stress, caused by oxygen, is the result of the metabolism of aerobic cells in the body. It is essential for the growth of all cells, regardless of the type of cell, but it can be dangerous if it is produced in excess [3]. These oxidative stresses can be eliminated through the body’s antioxidant system, such as SOD and GSH, but their imbalance causes the formation of excessive oxidative stresses to form free radicals that attack neuronal cells [17]. Consecutive oxidative stress can cause protein and DNA damage, leading to the apoptosis of neuronal cells, and ultimately can induce neurodegenerative diseases such as AD and Parkinson’s disease [3]. It was reported that persimmon had high ABTS, DPPH radical scavenging activities, and contained various catechins such as catechin, epicatechin and epigallocatechin (Table 1) [18]. In addition, the leaves of persimmon inhibit lipid peroxidation and exhibit a high level of flavonoid [19]. Based on these results, EFDK also has high antioxidant activity, which seems to reduce oxidative stress effectively.

The cause of ROS production is different in biological systems, among which ROS is produced during respiration and energy production through normal cell activity in mitochondria. ROS produced in mitochondria is cleared from the antioxidant system, but in excess it can cause apoptosis with a cell death program [2]. This cascade has been widely studied in hippocampal HT22 cells [20]. When oxidative stress is induced, cysteine intake is inhibited, resulting in the depletion of GSH. ROS accumulates about 50 to 100 times more than in the normal state, and calcium ion influx and cell death increase, since there are no glutamate receptors [21]. This accumulation of oxidative stress leads to the death of neurons, which ultimately causes cognitive dysfunction in the hippocampal region by blocking neurotransmission [4]. Consecutive oxidative stress can cause the consumption of antioxidants such as vitamins and various flavonoids. It has been reported that the various flavonoids present in EFDK can effectively scavenge ROS [19]. In the present study, it was confirmed that EFDK has a protective effect on hippocampal neurons with an in vitro analysis of HT22 cells (Figure 1).

The most prominent features of AD patients are apoptosis in the hippocampus, amygdala, and thalamus due to amyloid plaque and oxidative stress. These brain tissue damages indicate amnesia, cognitive impairment, and neurotransmitter abnormalities [22]. TMT shows loss of the hippocampal CA3 region of the mouse, indicating loss of memory impairment and pyramidal neurons, and inhibits the connection of synaptic circuits from CA3 to similar to AD by inhibiting the connections between hippocampal regions [12]. Therefore, behavioral assessment is one of the indicators used to assess the cognitive dysfunction that occurs in the early stages of AD [23]. To evaluate the spatial cognition and network between the hippocampus, frontal cortex, and cerebellum, Y-maze, passive avoidance and Morris water maze tests were evaluated (Figure 2, Figure 3 and Figure 4). These tests confirmed that EFDK intake improves spatial cognition, short-term memory, and long-term learning abilities in TMT-induced cognitive dysfunction. Long-term memory improvement was observed in mice fed persimmon leaves by evaluating the Morris water maze and step-down tests in Aβ_1-42_-induced animal model [24]. In addition, high molecular weight persimmon tannin has also been reported to improve long-term memory in mice induced by d-galactose [25]. Gallic acid in persimmon also improved short-term memory and long-term learning ability [26]. These results suggest that EFDK, which contains abundant flavonoids capable of scavenging oxidative stress, has been shown to improve cognitive dysfunction, which is a protective effect against behavioral and cognitive impairment, the early symptoms of AD.

In many studies, the brain has been identified as abnormally sensitive to external stress, and it is known that it is also easy for peroxidation of the brain membranes to occur [27]. Brain tissue contains fatty acids that are more sensitive to lipid peroxidation, and also not rich in antioxidants (about 10% compared to liver) [28]. Because of these conditions, brain tissue is considered to be sensitive to oxidative stress [27]. Oxidative stress can be eradicated by antioxidant substances, and increase the inflammatory response and lower levels of antioxidant enzymes in AD brain [29]. This causes the death of cranial nerve cells, the loss of neurotransmitters and neurotransmitter abnormalities, which can lead to AD beyond cognitive dysfunction [4]. Therefore, we studied the improvement of the antioxidant system with the consumption of EFDK in TMT-induced mice (Figure 5). The treatment of EFDK inhibited lipid peroxidation and up-regulated SOD and reduced GSH levels in TMT-induced mice. Leaves of persimmon containing various phenolic compounds such as quercetin, kaempferol and kaempferol derivatives, ameliorated the lipid peroxidation and antioxidant system [24]. Also, it was also reported that lipid peroxidation was inhibited by the improvement of lipid in rats fed persimmon peel and pulp [30]. Furthermore, it was reported that EFDK contains various flavonoids. These flavonoids have been shown to directly inhibit ROS in nerve cells, as well as to increase cysteine uptake, thereby preventing the reduction of GSH [31]. Similar to the antioxidant protection effects of leaf, pulp and peel intake, EFDK also protects brain tissue by protecting the antioxidant system with its antioxidant activity.

The cholinergic system is closely related to the amyloid mechanism that causes AD. Cholinergic neurotransmission is directly affected by Aβ [32]. Amyloid peptides inhibit neuronal transmission by inhibiting the absorption of choline in neurons and reducing the release of ACh from cells. It also has a high affinity for ACh receptors and forms a complex with the receptors to inhibit cascade neurotransmission [33]. In the central nervous system, AChE also forms a stable complex with Aβ to increase the rate of fibril formation and the neurotoxicity of Aβ fibril. This complex not only increases the IC_50_ value of tacrine, which is known to be an inhibitor of AChE, by about five to 10 times, but also exhibits strong neurotoxicity because its structure is very stable. [34]. Abnormalities of the cholinergic system lead to synapse loss, resulting in the death of neuronal cells, and ultimately cause the loss of cognitive function [35]. Based on these results, the activity of the cholinergic system was measured. EFDK improved the cholinergic system in mice with induced oxidative stress (Figure 6). (+)-Catechin, which is abundantly contained in persimmon, has been reported to inhibit the activity of AChE [36], and chlorogenic acid also showed AChE inhibitory activity in hippocampal and frontal cortex neurons [37]. These AChE inhibitory activities of persimmon containing these flavonoids potentially inhibit the degradation of ACh and may improve signal transduction. However, the activity of the Aβ-AChE complex and receptor needs to be further studied in future.

The abnormality of mitochondrial energy metabolism may affect the pathogenesis of AD, and damage to energy metabolism promotes aging by increasing the vulnerability of nerve tissue to excitotoxicity. The mitochondria, which are oxidative phosphorylation sites, react with four electrons and four hydrogen ions, and O_2_ is reduced to H_2_O to generate energy in the form of ATP [27]. However, when the function of the mitochondria is degraded by aging, electron leakage occurs in the electron transport chain [3]. This leads to abnormalities in intraneuronal glucose metabolism, and leads to depolarization by decreasing ATP-dependent membrane transport, which is essential for maintaining membrane potential [38]. This results in excessive production of ROS, resulting in cell excitotoxicity, and an excessive amount of glutamate reacts with neurons to become toxic [39]. This causes an abnormal function of the ion channel, causing excessive calcium ions to be introduced into the neurons, and the increase of calcium ions induces neurotoxicity, resulting in the death of the mitochondria [40]. In this process, the content of lactic acid is increased, and the increase of lactic acid stimulates Fe^2+^ and Fenton reaction to generate OH from H_2_O_2_. Calcium reacts with O^2−^ to activate nitric oxide synthetase, which can produce nitric oxide. Therefore, the reduction of energy metabolism and excitotoxicity by calcium ion is a mechanism to generate ROS, which can contribute to neural degeneration and death [39]. Based on these results, it was confirmed that the activity of mitochondria declined in TMT-induced mice, but the intake of EFDK decreased the production of ROS, and showed an increase in MMP and an increase in ATP content (Figure 7). In addition, caffeic acid and gallic acid, which are known to be abundant in persimmon, have been reported to protect cells by preventing intracellular Ca^2+^ influx [31]. These results suggest that EFDK improves the functional abnormality of mitochondria damaged by oxidative stress.

The most prominent feature of AD patients is the production and aggregation of Aβ. The aggregation of Aβ reacts with the mitochondrial membrane, producing oxidative stress, and causing an inflammatory reaction [41]. This inflammatory response increases the expression of TNF-α. Increased expression of TNF-α leads to a persistent inflammatory response, which promotes the phosphorylation of NF-kB, accelerating the production of cytokines and TNF-α [42]. TNF-α continuously promotes the phosphorylation of JNK. Abnormally activated p-JNK in neurons results in IRS-1pSer instead of tyrosine residues (IRS-1pTyr). Through this process, IRS-1pSer activates phosphoinositide 3-kinase (PI3K) which phosphorylates phosphatidylinositol (PI)-4,5-bisphosphate (PtdIns (4,5) P2) (PIP2) to PI-3,4,5-triphosphate (PtdIns (3,4,5) P3) (P1P3) [43]. This cascade continuously activates Akt by promoting the phosphorylation of Akt present in the inner surface of the plasma membrane. Comprehensively, IRS-1pTyr generally promotes the phosphorylation of Akt, thereby increasing cell proliferation and inhibiting apoptosis and thus enhancing cell survival [44]. Activated Akt in a phosphorylated form normally inhibits the activation of GSK-3β, and this inhibition blocks the phosphorylation of tau combining with tubulin and stabilizes microtubules. However, IRS-1pSer produced from p-JNK inhibits the phosphorylation of Akt. The decreased Akt activity does not block the activation of GSK-3β and promotes the phosphorylation of tau [45]. p-Tau binds itself to become a dimer, an oiligomer, and ultimately forms NFTs, which causes neuronal cell death [5]. In addition, normally activated Akt inhibits the activity of BAX and Bcl-2-associated death promoter (Bad), which promote the death of mitochondria, and increases the expression of Bcl-2 associated with mitochondrial survival. However, the reduced activation of Akt by the cascade of oxidative stress does not regulate these factors, and reduces mitochondrial activity [46]. The increased expression of BAX releases cytochrome c, which is involved in energy production in mitochondria to cytosol, and released cytochrome c reacts with pro-caspase-9 to form apoptosome. Increased cytosolic cytochrome c sequentially activates caspase-9 and caspase 3/7, resulting in apoptosis [41]. Finally, oxidative stress leads to apoptosis through a complex cascade, and ultimately leads to AD [27]. Based on these cascades, we studied how EFDK significantly reduced the expression of TNF-α, p-JNK, IRS-1pSer, p-tau, p-NF-κB, BAX and ratio of cytosolic/mitochondrial cytochrome c and increased the expression of p-Akt (Figure 8 and Figure 9). Vanillic acid, known to be abundant in the senses, increased the content of p-Akt and decreased the activity of GSK-3β in mouse models with amyloid beta-induced cognitive impairment. In addition, vanillic acid decreased the expression of p-NF-kB and BAX and inhibited neuronal cell death [47]. Chlorogenic acid also inhibited the phosphorylation of JNK, extracellular-signal-regulated kinase (ERK) 1/2, and p-38 and reduced the expression of p-NF-κB in LPS-induced inflammation models [48]. In addition, the leaves of persimmon decreased TNF-a, IL-1b, cyclooxygenase-2 (COX-2), and inducible nitric oxide synthase (iNOS) content in brain tissue by regulating inflammatory responses in APP/PS1 transgenic mice [49]. EFDK contains many flavonoids and has a protective effect on neuronal cells by regulating the JNK pathway and inhibiting apoptosis.

## 4. Materials and Methods 

### 4.1. Sample Preparation

The persimmon (*Diospyros kaki*) used in this experiment were purchased in March 2017 from a farm in Yeongam-gun, Korea, based on information obtained from the Division of Special Forest Products, National Institute of Forest Science. Samples were lyophilized using a vacuum tray drier (Operon, Gimpo, Korea) and the lyophilic zed samples were powdered and stored at −20 °C. The sample (60 g) was extracted with 50-fold 80% ethanol at 40 °C for 2 h. The extracted sample was filtered and concentrated using a vacuum rotary evaporator (N-N series, Eyela Co., Tokyo, Japan). The yield of the 80% ethanol extract was 36.34% of dried weight. This extracted sample (21.80 g) was re-suspended in 300 mL of distilled water, and successively fractionated with 300 mL of n-hexane, chloroform and ethyl acetate. Each fraction was re-lyophilized and powdered, and kept at −20 °C until use.

### 4.2. Total Phenolic and Flavonoid Contents

The total phenolic content (TPC) of four fractions of persimmon were assessed with Folin-Ciocalteu reagent [50]. Each sample were reacted with Folin-Ciocalteau reagent and Na_2_CO_3_, and incubated at room temperature for 2 h. The absorbance was measured at 760 nm using a spectrophotometer (Libra S32PC, Biochrom Ltd., Cambridge, UK). The results were expressed as mg gallic acid equivalent (GAE)/g. 

Total flavonoid content (TFC) analysis was performed by modifying the experimental method [51]. Diethylene glycol and 1 N NaOH were mixed with a sample, and the mixture was allowed to react at 30 °C for 60 min. The absorbance was measured at 420 nm. The measured absorbance was calculated by using a rutin-based calibration curve and the total flavonoid content was calculated.

### 4.3. Evaluation of Antioxidant Activity

Evaluation of antioxidant activity was conducted using 2,2′-azino-bis (3-ethylbenzothiazoline-6-sulfonic acid) (ABTS) and 1,1-diphenyl-2-picrylhydrazyl (DPPH) radical scavenging activities. A solution of ABTS in 100 mM phosphate buffer (pH 7.4) containing 150 M NaCl was added to 1.0 mM [azobis-(2-amidinopropane) HCl] (AAPH) and mixed and reacted in a water bath at 68 °C for 30 min. The prepared ABTS solution was diluted with distilled water to obtain an absorbance value of 0.70 ± 0.02 at 734 nm. The adjusted ABTS solution was mixed with the sample, and reacted at 37 °C for 10 min. The absorbance was measured at 734 nm [52].

DPPH radical scavenging activity was measured by dissolving 0.1 mM DPPH in 80% methanol and diluting this with 80% methanol to obtain an absorbance value of 1.00 ± 0.02 at 517 nm. 50 μL of the sample was mixed with 1.45 mL of the adjusted DPPH solution, reacted at room temperature for 30 min, and the absorbance was measured at 517 nm [53].

### 4.4. Lipid Peroxide

To measure the inhibitory effect of lipid peroxide using mouse brain tissue, brain tissue was harvested and homogenized by adding 20 mM Tris-HCl buffer (pH 7.4), and centrifuged at 12,000 g, for 15 min at 4 °C. The supernatant was used for the experiment [50]. Samples were mixed with brain tissue supernatant, 10 μM FeSO_4_, 0.1 mM ascorbic acid, and incubated at 37 °C for 1 h. After 1 h, 30% trichloroacetic acid and 1% thiobarbituric acid were added and heated in a water bath at 80 °C for 20 min, and the absorbance of the reaction solution was measured at 532 nm.

### 4.5. Cell Culture and Treatment

The HT22 cells derived from the brain hippocampal tissue of a mouse were used to study the characteristics of the brain neurons. It is a hippocampal-derived neuronal cell line that is used to study the characteristics of many neurodegenerative diseases such as AD and Parkinson’s disease [54]. HT22 cells were obtained in October 2017 from the Department of Anatomy of the College of Veterinary Medicine, Gyeongsang National University. HT22 cells were cultured in Dulbecco’s Modified Eagle’s Medium (DMEM) with 10% calf serum, 50 units/mL penicillin, and 100 μg/mL streptomycin at 37 °C in a humidified incubator containing 5% CO_2_.

### 4.6. Viability and Intracellular Reactive Oxygen Species (ROS)

Cells were spread on a 96-well plate (1 × 10^4^ cells/well), and after 24 h, the cells were treated with different concentrations of EFDK. MTT solution was added and reacted in an incubator. After 3 h, the cells were dissolved in DMSO and assayed at 570 nm in a microplate reader (Epoch 2, BioTek Instruments, Inc., Winooski, VT, USA). Cell viability was determined by the relative growth rate of cells. To measure the intracellular ROS, the same concentration of cells was spread on a black plate. After 24 h, samples were processed, and DCF-DA solution was added. Fluorescence was evaluated using a fluorescence microplate reader (Infinite 200, Tecan Co., San Jose, CA, USA) at 485 nm excitation and 530 nm emission filters [55].

### 4.7. Animals

**Figure 10:**
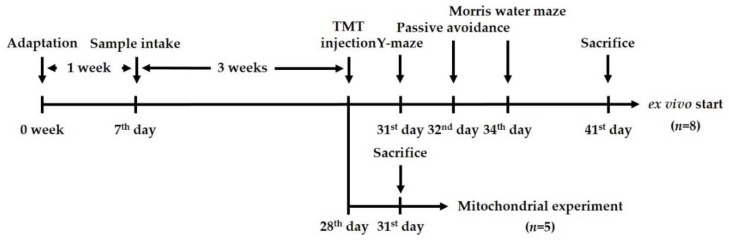
Experimental design of in vivo test for TMT (trimethyltin)-induced learning and memory impairment in mice.

The male ICR mice (4 weeks old) were purchased from an animal supplier (Samtako, Osan, Korea). For these experiments, the animals were divided to four groups: NC group (vehicle-intraperitoneally (i.p.) injected/vehicle-administration), TMT group (TMT-injected/vehicle oral administration), and EFDK 10 and EFDK 20 groups (TMT-injected/EFDK 10 and 20 mg/kg of body weight oral administration, respectively). The vehicle was used as 0.85% sodium chloride solution, and the injected TMT concentration was 7.1 μg/kg of body weight. Samples were taken directly into the stomach using the stomach tube. All animal experiments received the approval of the Animal Care and Use Committee of Gyeongsang National University (certificate: GNU-170605-M0023, 05062017), and were carried out according to the provisions of the Policy of the Ethical Committee of the Ministry of Health and Welfare, Republic of Korea. The experiment design is presented in Figure 10.

### 4.8. Behavioral Tests

Three days after the i.p. injection of TMT, behavioral estimations were investigated in a time sequence. A Y-maze test was used to measure spontaneous alternation behavior. This estimation was performed 3 days after TMT injection. A Y-maze consists of Y-shaped arms divided into A, B and C (length 90 cm × width 12 cm height). The mouse was placed at the end of one arm, and allowed to move freely for 8 min. Movement was recorded with a tracking system (Smart 3.0, Panlab, Barcelona, Spain). The number of arm entries of a mouse is defined by the following equation [56]:

Alternation behavior = [(number of alternations)/(total arm entries − 2)] × 100.

The passive avoidance test was performed using a shuttle box device. The device consisted of a grid floor divided into a bright place and a dark place. After placing the animal on one side and turning on the light and opening the passage, the door on the connecting path was closed and electric shock (0.5 mA) was applied for 3 s while the animal was completely in the dark area. After 24 h, the time spent in the bright area was measured to confirm memory formation and fear learning [57].

A Morris water maze test was performed to evaluate cognitive memory and spatial learning ability. A circular water pool (diameter 90 cm, height 30 cm) filled with water was divided into quadrants. The water in the pool was diluted with squid ink and a black platform 1 cm below the surface of the water was locked on one side of the quadrant. While the animals were swimming, they were trained repeatedly four times a day, each time with different positions, and the tracing path was recorded with Smart 3.0 (Panlab) (hidden trials). After a four-day training trial, the time the experimental animals stayed in the area where the platform was located (probe trials) was recorded [58].

### 4.9. Plasma FRAP

Mouse blood was obtained from the postcaval vein and stored in a heparin tube. The blood was centrifuged at 10,000 g for 10 min at 4 °C to obtain plasma, and used to measure plasma ferric reducing/antioxidant power (FRAP). Plasma and TPTZ solution [300 mM sodium acetate buffer (pH 3.6), 10 mM TPTZ in 40 mM HCl and 20 mM FeCl] were mixed for 15 min at 37 °C, and the absorbance of the reaction solution was measured at 593 nm [59].

### 4.10. Brain Tissue Preparation

After cognitive behavioral testing was completed, the mice were fasted for 12 h and sacrificed by CO_2_ anesthesia for ex vivo experiments. The mouse brain was harvested and homogenized with a bullet blender (Next Advance Inc., Averill Park, NY, USA) with 10 volumes of phosphate-buffered saline (PBS) for acetylcholine (ACh), acetylcholinesterase (AChE), malondialdehyde (MDA), and SOD assays, and with 10 volumes of 10 mM phosphate buffer containing 1 mM EDTA (pH 6.7) for the reduced GSH assay at 4 °C. The Bradford method was used to measure the protein used in the experiment [60].

### 4.11. Lipid Peroxidation

The brain tissue centrifuged at 2500 g for 10 min was reacted with 1% phosphoric acid and 0.67% thiobarbituric acid at 95 °C for 1 h in a water bath. The absorbance of the supernatant was measured at 532 nm. After centrifugation at 2500 g for 10 min, the supernatant was used for the experiment [50].

### 4.12. Superoxide Dismutase (SOD) Level

The brain tissue was centrifuged at 10,000× *g* for 30 min, and the supernatant was discarded to take the pellet. The pellet was vortexed with 1 × cell extraction buffer (Dojindo Molecular Technologies, Inc., Rockville, MD, USA) for 30 min in 5 min increments. After this mixture was centrifuged at 400× *g* for 10 min, the supernatant was used for the experiment. SOD content was assessed using a SOD determination kit (Sigma-Aldrich Chemical Co., St. Louis, MO, USA), expressed as SOD (U/mg of protein).

### 4.13. Reduced Gluthathione (GSH) Contents

The brain tissue pretreated with phosphate buffer was centrifuged at 10,000× *g* for 15 min, and the supernatant obtained was used for reduced GSH contents. This supernatant was reacted with the same volume of 30% metaphosphoric acid to precipitate an interference-inhibiting protein at 2000× *g*. The supernatant was reacted with 0.26 M tris-HCl (pH 7.8), 0.65 N NaOH, 1 mg/mL of phthaldialdehyde, and the reaction was allowed to proceed at room temperature for 15 min. Then, the fluorescence intensity was measured using a fluorescence microplate reader (Infinite 200) at a wavelength of 320 nm (excitation filter) and 420 nm (emission filter). The reduced GSH content was assigned to a standard curve [61].

### 4.14. Evaluation of Cholinergic System

The brain tissue pretreated with PBS was centrifuged at 14,000× *g* for 30 min, and the supernatant was used for the experiment. To evaluate AChE activity, the supernatant and 50 mM sodium phosphate buffer were mixed and pre-incubated at 37 °C for 15 min, and then Ellman’s reaction mixture was added to the reaction mixture. The absorbance wavelength was measured at 405 nm, and the activity of the enzyme was expressed as % activity relative to the NC group [62]. To measure ACh content, the same supernatant was reacted with alkaline hydroxylamine reagent [3.5 N sodium hydroxide and 2 M hydroxylamine in HCl], and allowed to stand at room temperature for 1 min. Then, 0.5 N HCl, 0.37 M FeCl_3_ in 0.1 N HCl were reacted, and the absorbance was measured at a wavelength of 540 nm [63].

### 4.15. Cerebral Mitochondrial Extraction

Mouse brain tissue was homogenized using mitochondria isolation (MI) buffer [215 mM mannitol, 75 mM sucrose, 0.1% BSA, 20 mM HEPES sodium salt (pH 7.2)] containing 1 mM EGTA. After the homogenized tissue was centrifuged at 1300× *g* for 10 min at 4 °C, the supernatant was centrifuged again at 13,000× *g* for 10 min. The centrifuged pellet was mixed with MI buffer containing 0.1% digitonin, and the mixture mixed with MI buffer containing 1 mM EGTA was centrifuged at 13,000× *g* for 15 min at 4 °C. Next, the pellet was mixed with the MI buffer, and centrifuged again at 10,000× *g* for 10 min. The final pellet was mixed with the MI buffer to conduct the experiment.

### 4.16. Estimation of Mitochondrial Function

To assess the mitochondrial intracellular ROS, a DCF-DA assay was conducted. The isolated mitochondria were reacted with DCF-DA for 20 min with KCl-based respiration buffer (125 mM potassium chloride, 2 mM potassium phosphate, 20 mM HEPES, 1 mM magnesium chloride, 500 μM EGTA, 2.5 mM malate and 5 mM pyruvate), and the fluorescence was measured using a fluorescence microplate reader (Infinite 200) at 485 nm excitation and 530 nm emission filters [64].

To evaluate MMP, the mitochondrial extract mixed with MI buffer containing 5 mM malate and pyruvate was gently reacted to JC-1 in a black 96-well plate. The plate was incubated in a dark room for 20 min at room temperature, and fluorescence was measured using a fluorescence microplate reader (Infinite 200) at 530 nm excitation and 590 nm emission filters [64].

ATP content was measured using an ATP bioluminescence assay kit (Sigma-Aldrich Chemical Co.) according to the manufacturer’s protocol. The ATP contents of extracted mitochondria were determined using the oxidation value of luciferase, which was determined by using the value of luciferin, which is oxidized when catalyzed by luciferase. The ATP content was calculated according to a standard curve.

### 4.17. Western Blot Analysis

To evaluate the expression of proteins, Western blot analysis was performed by homogenizing the brain tissue with lysis buffer (GeneAll Biotechnology, Seoul, Korea) containing 1% protease inhibitors. These homogenates were centrifuged for 10 min at 13,000× *g* at 4 °C. After the concentration of protein was equally adjusted, the protein was separated by electrophoresis using sodium dodecyl sulfate polyacrylamide gel electrophoresis (SDS-PAGE). The proteins were transferred to a polyvinylidene difluoride (PVDF) membrane (Millipore, Billerica, MA, USA), and the membranes were blocked with 5% skim milk to reduce the interference effects of other proteins. After incubation for 12 h with a primary antibody (1:1000) containing AChE, p-JNK, p-tau, cytochrome c, IRS-1pSer and β-actin (Santa Cruz Biotechnology, Dallas, TX, USA) and TNF-α, p-Akt, p-NF-κB, BAX (Cell Signaling Technology, Danvers, MA, USA), and secondary antibody (1:1000, Cell Signaling Technology) it was reacted at room temperature for 1 h. The protein expression was detected using a Western blot imager (iBright Imager, Thermo-Fisher Scientific, Waltham, MA, USA), and the protein densities were calculated using image making software (ImageJ software, National Institutes of Health, Bethesda, MD, USA).

### 4.18. Statistical Analysis

All experimental results were presented as mean ± standard deviation (SD). Statistically significant differences between the groups were indicated by one-way analysis of variance (ANOVA). Significant differences were identified using Duncan’s new multi-range test (*p* < 0.05) with the SAS program (version 9.4, SAS Institute Inc., Cary, NC, USA).

## 5. Conclusions

**Figure 11:**
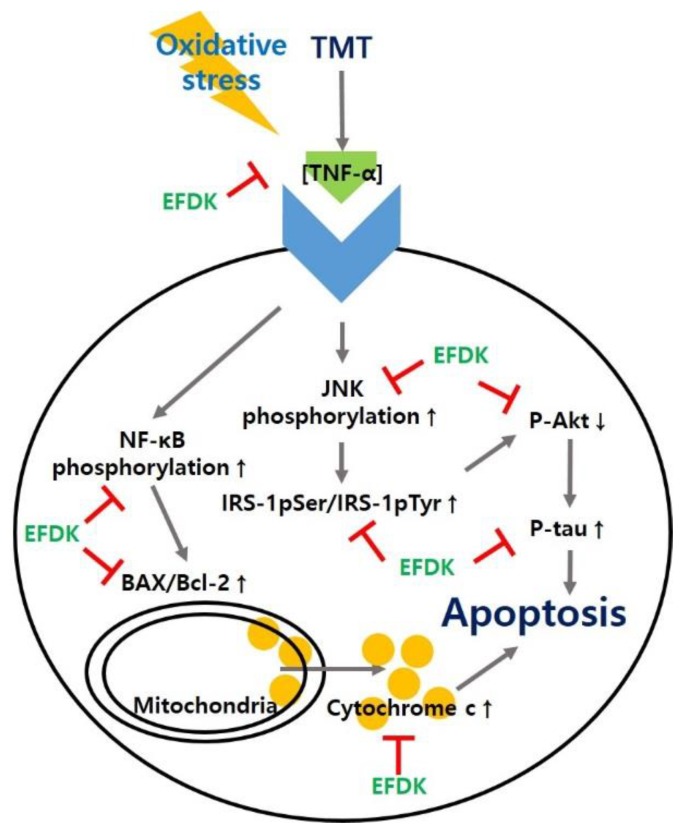
A schematic illustration shows the ameliorating effect of ethyl acetate fraction from persimmon (EFDK) in TMT-induced behavioral and memory dysfunction in ICR mice via regulation of the JNK/Akt signaling and apoptotic pathways.

In summary, based on this research, EFDK had a high concentration of total phenolic and flavonoid content with excellent in vitro antioxidant activity and hippocampal neuronal cell protective effect. Also, EFDK considerably improved the spontaneous alternation behavior, short-term memory and spatial learning acquisition, and long-term memory of TMT-induced mice. EFDK increased the blood antioxidant capacity and significantly protected the antioxidant system and cholinergic system in brain tissue. Furthermore, it also ameliorated mitochondrial function by regulating the expression of protein signaling via JNK/Akt and apoptotic pathways (Figure 11).

We have confirmed the ameliorating effects of EFDK on oxidative stress and cholinergic system damage in this study. Thus, we will carry out the improvement of EFDK against neurotoxicity and neuro-inflammation using an amyloid beta_1-42_-induced mouse model.

## Figures and Tables

**Table 1:** Antioxidant capacity of ethyl acetate fraction from persimmon (EFDK).

TPC ^a^	TFC ^b^	ABTS ^c^	DPPH ^d^	MDA ^e^
81.75 ± 1.52	187.52 ± 3.21	25.03	35.47	389.21

^a^ TPC, total phenolic content; ^b^ TFC, total flavonoid content; ^c^ ABTS, ABTS radical scavenging activity; ^d^ DPPH, DPPH radical scavenging activity; ^e^ MDA, malondialdehyde (MDA) inhibitory effect. Results are mean ± standard deviation (SD) (*n* = 3). Results of TPC and TFC are presented as mg of GAE/g and mg of RE/g, respectively. Results of ABTS, DPPH and MDA are presented as IC_50_ value (μg/mL).

## Supplementary Materials

The following are available online at http://www.mdpi.com/1422-0067/19/5/1499/s1.

## Abbreviations

AD	Alzheimer’s disease
ROS	reactive oxygen species
NFT	neurofibrillary tangles
TMT	trimethyltin
EFDK	ethyl acetate fraction from persimmon
TPC	total phenolic contents
TFC	total flavonoid contents
MDA	malondialdehyde
SOD	superoxide dismutase
GSH	reduced glutathione
AChE	acetylcholinesterase
ACh	acetylcholine
MMP	mitochondrial membrane potential
JNK	c-Jun N-terminal kinase
Akt	protein kinase B
TNF-α	tumor necrosis factor-alpha
IRS-1pSer	phosphorylated insulin receptor substrate 1
NF-κB	nuclear factor kappa-light-chain-enhancer of activated B cells
BAX	Bcl-2-associated X protein
Aβ	amyloid-beta
