# Supplementary material for: Ethyl Acetate Fraction from Persimmon (Diospyros kaki) Ameliorates Cerebral Neuronal Loss and Cognitive Deficit via the JNK/Akt Pathway in TMT-Induced Mice
Source: Int J Mol Sci. 2018 May 17;19(5):1499. doi: 10.3390/ijms19051499 (PMC5983595; doi:10.3390/ijms19051499)
Supplement: Supplementary file 1 [file ijms-19-01499-s001.zip › ijms-301505-SI.pdf]

Table 1. Ascorbic aicd contents of five cultivars of *Diospyros kaki*. **Unit: mg/100 g**

	**Dungsi**	**Godongsi**	**Gojongsi**	**Gabjubaekmok**	**Bansi**
**Vitamin C**	206.17±6.87[a]	135.54±17.90[b]	106.72±2.03[d]	116.02±11.97[c]	89.64±1.21[e]

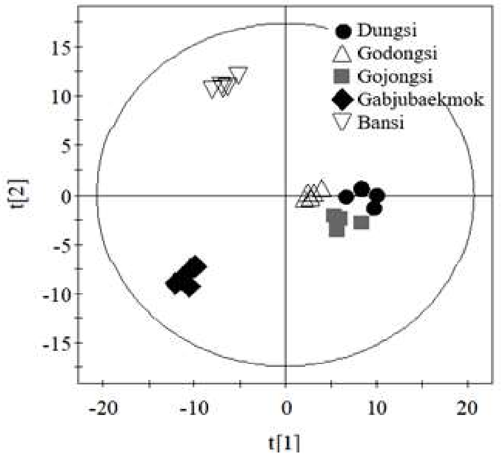

Figure 1. PLS-DA score scatter plot of five cultivars of *Diospyros kaki*. Results shown are mean ± SD (n=5). Data were statistically considered at $p<0.05$, and different small letters represent statistical differences.

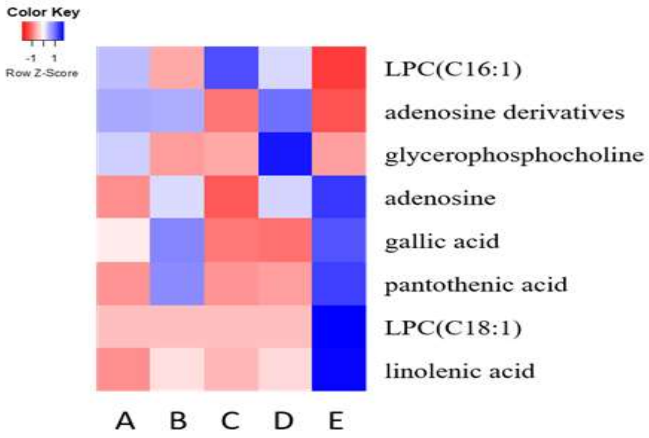

Figure 2. Heatmap of the identified metabolites of five cultivars of *Diospyros kaki*. Results shown are mean ± SD (n=5). Data were statistically considered at $p<0.05$, and different small letters represent statistical differences. A: Dungsi, B: Godongsi, C: Gojongsi, D: Gabjubeakmok, E: Bansi.

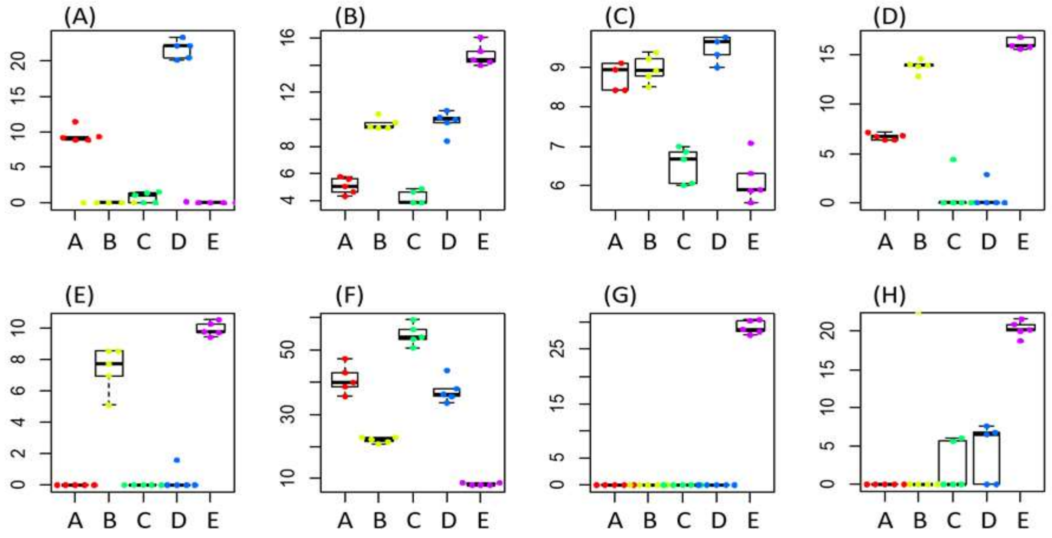

Figure 3. Fold change of identified metabolites of five cultivars of *Diospyros kaki*. (A): glycerophosphocholine, (B): adenosine, (C): adenosine derives, (D): gallic acid, (E): pantothenic acid, (F): lysophosphatidylcholine (LPC, C16:1), (G): lysophosphatidylcholine (LPC, C18:1), (H): linolenic acid. Results shown are mean ± SD (n=5). Data were statistically considered at $p<0.05$, and different small letters represent statistical differences. A: Dungsi, B: Godongsi, C: Gojongsi, D: Gabjubaekmok, E: Bansi.

Our previous study was conducted to compare nutritional and metabolic analysis between the ethanol extract of five persimmon cultivars (Korean name: Dungsi, Godongsi, Gojongsi, Gabjubaekmok and Bansi). The results of vitamin C content using HPLC analysis showed averagely 130 mg/100 g (89.64-206.17mg/100 g) (Table 1). In addition, metabolite analysis of each cultivar was carried out using UPLC-IMS-QTOF/MS^2. Using the PLS-DA score scatter plot, it was confirmed that the metabolites among cultivars showed significantly different (Figure 1). To determine the significant difference of these substances, metabolic analysis was also carried out (Figure 2 & 3). Diverse metabolites such as lysophosphatidylcholine (LPC, C16:1), lysophosphatidylcholine (LPC, C18:1), adenosine, adenosine derives, glycerophosphocholine, gallic acid, pantothenic acid and linolenic acid identified through metabolite analysis showed significant difference between five cultivars. The relative differences and fold change of the compounds showing differences between varieties were presented through heatmap analysis (Figure 2) and box plot (Figure 3).
